# The Diagnosis and Treatment of Hæmorrhage in Typhoid Fever

**Published:** 1909-02-27

**Authors:** C. Willett Cunnington


### The General Practitioner's Column.

[Contributions to this Column are invited, and if accepted will be paid for.

### THE DIAGNOSIS AND TREATMENT OF HÆMORRHAGE IN TYPHOID FEVER.

By C. WILLETT CUNNINGTON, M.B., B.C. (Cantab.).

There is, perhaps, no more searching test of a doctor's skill, promptitude and resourcefulness than the diagnosis and treatment of a case of typhoid fever in which severe hæmorrhage is just taking place. Considering the immense difficulty of the situation into which the doctor is suddenly plunged, he will be wise to have certain methods to be employed ready formularised in his mind; while the weapons needed should be ready, as it were, at the bedside.

We can disregard the initial hæmorrhages occurring in the first week of typhoid fever; these are slight in amount and never dangerous. Far otherwise are the bleedings which may occur in the third week when the sloughs are separating. The most "popular" day for serious hæmorrhage is the eighteenth of the disease.

There are certain small signs which may be observed a few hours before the more alarming symptoms arise; thus, some importance should be attached to unconscious passage of urine. The cause of this "danger signal" is possibly a momentary disturbance of the vaso-motor centres due to a bleeding slight enough to induce a wave of cerebral anæmia; the incontinence resembles, in fact, that seen sometimes in attacks of syncope. Slight restlessness, otherwise unexplainable, should likewise be regarded as suspicious.

It is desirable to bear in mind that, though many hæmorrhages are at once copious, producing sudden and obvious symptoms of collapse, yet often the bleeding begins slowly and causes a succession of signs only gradually becoming marked. In these cases a falling temperature is perhaps the first point noticeable, occurring before the pulse rate has increased. There can be no bleeding going on while the temperature remains high.

Respirations become more frequent and shallow, accompanied by increasing frequency of pulse rate. At this stage patients with diarrhœa may begin to pass blood per rectum, but where there is constipation the blood may not pass for some hours. A gurgling sound in the intestines is suggestive, especially in constipated cases, since these are the more liable of the two to hæmorrhage.

Where enough blood has been lost, the patient becomes blanched and restless and unconscious, with a cold sweat on face and hands. For a few minutes the observer may be uncertain whether this condition is one of hæmorrhage or of sudden myocardial failure. The thermometer should be his guide. Yet one often sees a fatal hesitancy, during which stimulants are given to "help the heart," and, incidentally, to help the bleeding.

In treating these cases it is essential that the medical attendant should have quite clear in his mind the steps he intends to take. To become frightened and waver is disastrous. The doctor who loses his head speedily loses his patient as well. Morphia, say, $\frac{1}{4}$ gr. to $\frac{1}{3}$ gr., should be injected hypodermically, at once. There can be no exception to this rule. The "starch and opium" enema of the text-books is of doubtful use. Valuable time is lost in its preparation and administration, and there is the risk of a bloody stool washing it, the next moment, out into the bed. The drug must be introduced into the circulation without delay, and there is no quicker means than by the hypodermic needle.

While bleeding is, presumably, still going on, the treatment resolves itself into four things only:—push the morphia; raise the foot of the bed at least two feet; remove pillows; and banish relatives from the sick-room. This latter point is sound therapeutics. The administration of stimulants while hæmorrhage continues indicates panic. After these points have been effected the doctor waits with his finger on the pulse. To decide when the bleeding has stopped is difficult enough, yet he must do so. Probably the pulse-rate ceasing to increase is the best guide. But suppose the bleeding seems to continue and the case is nearly desperate? If the patient can swallow, a dram of ol. terebinthini in mucilage should be given, and repeated in half an hour if necessary. It is reasonable to lower the blood-pressure to a minimum with inhalations of amyl nitrite, but meanwhile the morphia syringe must be used absolutely fearlessly.

If by these means the bleeding is stopped the picture changes somewhat; life still exists, but at a very low ebb; the pulse rate is extremely rapid; the respirations are shallow, frequent and irregular; the body is cold and clammy. The problem now is to maintain the heart's action without dislodging the clot from the opened vessel, a problem needing the nicest judgment. The little blood remaining in the body is needed for the vital organs, especially the brain, and this is encouraged by extreme tilting of the bed, at an angle even of forty-five degrees.

For the process of "keeping the heart going," two factors are necessary: one, a sufficient blood-pressure for the organ to beat against; the other, a sufficient stimulation of its nerves and muscle fibres. The former point is apt to be overlooked. In order to give sufficient pressure for the pump action to continue, there is nothing so useful as the hypodermic injection of supra-renal extract. An amount equivalent to five minims of the liquid extract is a good dose. This acts better than strychnine and may be pushed where strychnine fails to do more than induce muscular rigidity; moreover, excess of strychnine will sometimes increase the shallowness and irregularity of the breathing.

The question of raising the blood-pressure suggests the use of a saline infusion, either subcutaneously or intravenously. The danger, however, of diluting the blood and diminishing its coagulability is great; this, and the difficulty of controlling the increased pressure, makes it of doubtful value. It is of much greater use, after forty-eight hours, if the patient has difficulty in swallowing enough fluid; it then combats a threatened collapse without risk of renewed hæmorrhage.

For stimulation of the heart and its nerves we have, of course, strychnine and ether. Here I would put in a good word for musk. It is useful to keep five grains in a small bottle covered with a drachm or two of absolute alcohol. Twenty minims of the alcohol can be used hypodermically and a little fresh spirit added to the "stock bottle." After a few minutes' shaking and standing a reasonably strong "home-made" tincture is ready, and in this way none of the precious drug is wasted. In a bad case it is immensely stimulating to the enfeebled heart. External stimulants such as hot bottles round the head and thorax must not be forgotten, and a mustard poultice to the præcordium may be tried. The application of ice to the iliac fossæ is a questionable proceeding. I conceive it might even do harm.

The nursing arrangements in a case of this sort are of extreme importance. No matter how competent the nurses may be, it is desirable to superintend their doings. A policy of "masterly inactivity" is called for, the patient being left unmoved for forty-eight hours; all motions must be allowed to remain in the draw sheet and no sort of cleansing should be permitted.

The relatives may clamour that the patient should be given food "to keep up his strength." It is, however, a good rule to give nothing but morphia hypodermically for forty-eight hours. At the end of that time small sips of water with perhaps a little whey will tide over the third day. After seventy-two hours the amount may be increased, but it is better to give nothing that can leave a solid residue in the intestines until the bowels have been emptied of the contained blood clot. It is best to leave the patient constipated for a week; and, until the colon is clear, milk, which forms considerable fæcal matter, should be stopped. Hence the value of whey.

The medical attendant must assure the friends that a patient can exist for several days at an extremely low level of vitality, and in these cases of bad hæmorrhage we must try to hold the balance poised between the two dangers—a second hæmorrhage and heart failure. Of the two the former is the more to be feared, as a second severe bleeding occurring within eighteen hours of the first is almost always fatal.

